# Loss of cerebellar glutamate transporters EAAT4 and GLAST differentially affects the spontaneous firing pattern and survival of Purkinje cells

**DOI:** 10.1093/hmg/ddy169

**Published:** 2018-05-08

**Authors:** Emma M Perkins, Yvonne L Clarkson, Daumante Suminaite, Alastair R Lyndon, Kohichi Tanaka, Jeffrey D Rothstein, Paul A Skehel, David J A Wyllie, Mandy Jackson

**Affiliations:** 1 The Centre for Discovery Brain Sciences, The University of Edinburgh, Hugh Robson Building, Edinburgh, UK; 2 School of Energy, Geoscience, Infrastructure and Society, Heriot-Watt University, John Muir Building, Riccarton, Edinburgh, UK; 3 Laboratory of Molecular Neuroscience, Medical Research Institute, Tokyo Medical and Dental University, Bunkyo-Ku, Tokyo, Japan; 4 Department of Neurology and Neuroscience, Johns Hopkins University, School of Medicine, Baltimore, MD, USA; 5 Centre for Brain Development and Repair, Institute for Stem Cell Biology and Regenerative Medicine, Bangalore, India

## Abstract

Loss of excitatory amino acid transporters (EAATs) has been implicated in a number of human diseases including spinocerebellar ataxias, Alzhiemer’s disease and motor neuron disease. EAAT4 and GLAST/EAAT1 are the two predominant EAATs responsible for maintaining low extracellular glutamate levels and preventing neurotoxicity in the cerebellum, the brain region essential for motor control. Here using genetically modified mice we identify new critical roles for EAAT4 and GLAST/EAAT1 as modulators of Purkinje cell (PC) spontaneous firing patterns. We show high EAAT4 levels, by limiting mGluR1 signalling, are essential in constraining inherently heterogeneous firing of zebrin-positive PCs. Moreover mGluR1 antagonists were found to restore regular spontaneous PC activity and motor behaviour in EAAT4 knockout mice. In contrast, GLAST/EAAT1 expression is required to sustain normal spontaneous simple spike activity in low EAAT4 expressing (zebrin-negative) PCs by restricting NMDA receptor activation. Blockade of NMDA receptor activity restores spontaneous activity in zebrin-negative PCs of GLAST knockout mice and furthermore alleviates motor deficits. In addition both transporters have differential effects on PC survival, with zebrin-negative PCs more vulnerable to loss of GLAST/EAAT1 and zebrin-positive PCs more vulnerable to loss of EAAT4. These findings reveal that glutamate transporter dysfunction through elevated extracellular glutamate and the aberrant activation of extrasynaptic receptors can disrupt cerebellar output by altering spontaneous PC firing. This expands our understanding of disease mechanisms in cerebellar ataxias and establishes EAATs as targets for restoring homeostasis in a variety of neurological diseases where altered cerebellar output is now thought to play a key role in pathogenesis.

## Introduction

Excitatory amino acid transporter 4 (EAAT4) and GLAST/EAAT1 (rodent/human nomenclature) are members of a family of five sodium-dependent plasma membrane glutamate transporters (EAATs) (1) and are the major glutamate transporters in the cerebellum, the region of the brain essential for maintaining postural control and coordination of voluntary muscle movement (2). Various studies have implicated loss of EAAT4 and/or GLAST/EAAT1 in the pathogenesis of several disorders affecting the motor system including several subtypes of spinocerebellar ataxia (SCA), SCA1 (3,4), SCA5 (5–7), SCA7 (8), episodic ataxia type 6 (9–11), spinal muscular atrophy (12) and fragile X associated tremor/ataxia syndrome (13). Furthermore, disrupted GLAST/EAAT1 function has been associated with schizophrenia (14,15) and cerebellar dysfunction is now also being linked to the pathophysiology of Alzheimer’s disease (16,17), autism (18–20) and other cognitive and neuropsychiatric disorders (21–23).

EAATs are responsible for the maintenance of low extracellular glutamate levels which prevents neurotoxicity whilst ensuring accurate synaptic communication (1). However, the full detail of the pathogenic effects of transporter loss in disease states is not yet clear and evidence is lacking for a primary role of EAAT4 loss in cerebellar ataxia.

EAAT4 is expressed in Purkinje cells (PCs) (24), the principal neurons and sole output of the cerebellar cortex, whereas GLAST/EAAT1 is the predominant glial transporter, being expressed in Bergmann glia 6-fold more abundantly than GLT-1/EAAT2 (25). GLAST/EAAT1 is believed to be present uniformly throughout the cerebellum in functional excess (26). In contrast, EAAT4 displays a differential pattern of expression within parasagittal bands, mapping onto that of aldolase C (zebrin II), with zebrin-positive (Z+) PCs expressing EAAT4 to a much higher level than zebrin-negative (Z–) PCs (27,28). This variable EAAT4 density has been shown to have synaptic physiological consequences with larger glial AMPAR-mediated current amplitudes observed in regions where PCs have lower endogenous levels of EAAT4 (29) and the synaptic activation of mGluR1a, which exhibits the same peri-synaptic distribution as EAAT4 (28,30), found to be dampened in regions of high EAAT4 expression despite no differential expression of mGluR1a (31). This physiological effect of EAAT density on synaptic activity is due to the slow glutamate translocation time of EAATs with multiple cycles of transport not effective in the rapid removal of glutamate from the synaptic cleft (32). Instead it is the density of EAATs that governs the rapidity of binding and sequestration of glutamate after synaptic release (33).

PCs, in addition to receiving excitatory and inhibitory synaptic input, also exhibit spontaneous high frequency repetitive firing (34,35), the frequency of which was recently reported to vary between Z+ and Z– bands of the cerebellar cortex in rats (36) and mice (37). To investigate whether a pathological loss of EAAT4 and GLAST/EAAT1 protein has an effect on the spontaneous activity of PCs, and whether such a defect plays a key role in the pathogenesis of cerebellar ataxia, we performed whole-cell patch clamp recordings of PCs *in vitro* in EAAT4 (ET4^–^^/^^–^) and GLAST (GLAST^–/–^) knockout mice. Here, we report that loss of EAAT4 enhances the spontaneous firing frequency and decreases the regularity of action potential firing of Z+ PCs by a mechanism mediated through mGluR1 signalling. In contrast, the intrinsic activity of Z– PCs is reduced in mice lacking GLAST and this is mediated by NMDA receptor signalling. Motor deficits arising from loss of EAAT4 and GLAST function are alleviated by mGluR1 and NMDA receptor blockade, which restore normal spontaneous simple spike activity in ET4^–/–^ and GLAST^–/–^ knockout mice respectively. Furthermore, loss of EAAT4 and GLAST results in loss of Z+ and Z– PCs, respectively. Thus, this study demonstrates that within the cerebellum, plasma membrane glutamate transporters have differential roles in regulating the intrinsic firing frequency and survival of PCs, both key elements in normal cerebellar function by ensuring accurate and efficient processing of sensory information. These data refine our understanding of how glutamate transporter dysfunction can disrupt the encoding of information within discrete functional units and contribute to disease pathogenesis in cerebellar disorders.

## Results

### Differential effect of cerebellar transporter loss on the spontaneous firing frequency of PCs

**Figure 1.**
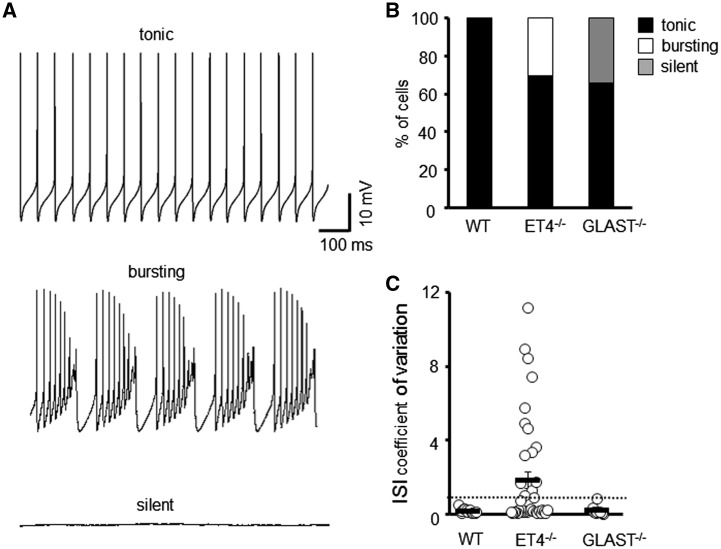
Different modes of intrinsic PC firing in EAAT4 and GLAST knockout mice. (**A**) Sample tonic, bursting and silent traces of PC current clamp recordings. (**B**) Ratio of WT (*N* = 5, *n* = 26), ET4^–/–^ (*N* = 6, *n* = 48) and GLAST^–/–^ (*N* = 4, *n* = 23) PCs that fire tonically, burst fire or are silent. (**C**) Coefficient of variation of ISI for WT, ET4^–/–^ and GLAST^–/–^ cells. Dotted line depicts CV = 1.

To elucidate what effect reduced levels of the two predominant cerebellar glutamate transporters have on the intrinsic properties and spontaneous firing pattern of PCs, we carried out *in vitro* whole-cell patch clamp recordings on PCs from ET4^–/–^ and GLAST^–/–^ mice. All recordings were carried out in the presence of AMPA and GABA_A_ receptor antagonists to block fast synaptic inputs. This revealed heterogeneity within the pattern of spontaneous simple spike activity (Fig. 1A and B). All wild-type PCs possessed a regular (tonic) rate of action potential firing, but 30% of PCs in ET4^–/–^ mice were observed to fire irregularly with periods of high frequency activity (bursting), although regular tonic firing could be restored in all bursting cells by delivery of a negative current. The regularity of simple spike activity was quantified by calculating the coefficient of variation of inter-spike interval (CV ISI) with a value >1 indicating high variance/irregularity (Fig. 1C) and used to classify whether a PC was burst firing. In contrast, 34% of PCs in GLAST^–/–^ mice were found to be silent with no spontaneous action potential firing recorded within a minimum of 2 min. However, action potentials could be elicited in all silent cells by a depolarizing current step. The remaining PCs in ET4^–/–^ and GLAST^–/–^ mice (70 and 66%, respectively) exhibited tonic firing with a CV ISI < 1.

### EAAT4 modulates the firing pattern of zebrin II positive (Z+) PCs whereas GLAST modulates the firing pattern of zebrin II negative (Z–) PCs

Given EAAT4 expression levels differ through the cerebellum (27,28), we next determined whether the heterogeneity in firing observed in ET4^–/–^ and GLAST^–/–^ knockout mice correlated with EAAT4 expression profile. This was achieved by creating ET4^–/–^-eGFP and GLAST^–/–^-eGFP mice by crossing ET4^–/–^ and GLAST^–/–^ mice with a fluorescent reporter BAC mouse (eGFP) in which eGFP cDNA is under the control of the EAAT4 promoter (38).

**Figure 2.**
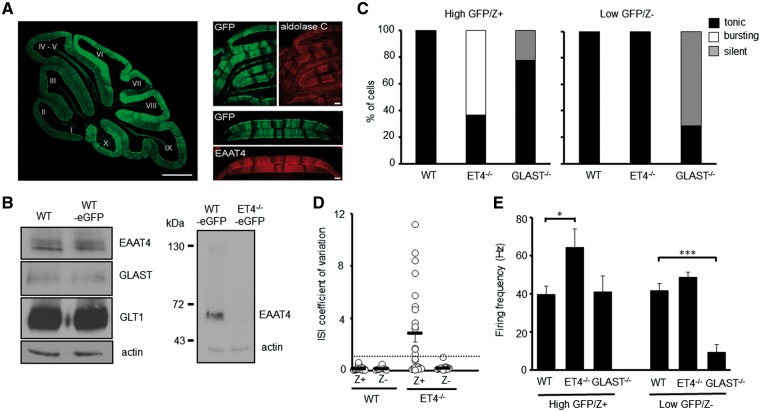
Modes of intrinsic PC firing correlate with differential expression profiles of EAAT4. (**A**) Left, mid-sagittal cerebellar section from a fluorescent reporter BAC mouse in which eGFP (green) cDNA is under the control of the EAAT4 promoter. Bar, 500 µm. Right, coronal cerebellar sections from WT-eGFP (green) reporter mouse immunostained for either aldolase C (red) or EAAT4 (red). Bar, 50 µm. (**B**) Immunoblot analyses of EAAT protein levels in cerebellar homogenates from WT, WT-eGFP and ET4^–/–^-eGFP mice. (**C**) Ratio of high GFP (Z+) and low GFP (Z–) expressing PCs in WT-eGFP (*n* = 35), ET4^–/–^-eGFP (*n* = 33) and GLAST^–/–^-eGFP (*n* = 16) mice that fire tonically, burst fire or are silent. (**D**) Coefficient of variation of ISI for WT-eGFP and ET4^–/–^-eGFP PCs. Dotted line depicts CV = 1. (**E**) Spontaneous firing frequency of high GFP (Z+) and low GFP (Z–) expressing PCs in WT-eGFP, ET4^–/–^-eGFP and GLAST^–/–^-eGFP mice. Mean ± SEM.

To confirm the fluorescent intensity of GFP accurately reflects EAAT4 promoter activity, we first visualized mid-sagittal cerebellar sections from eGFP reporter mice. This revealed a higher GFP signal in posterior lobules VI–X compared with anterior lobules I–V (Fig. 2A) consistent with the fact EAAT4 expression is higher in posterior lobules. Next, coronal cerebellar sections from eGFP reporter mice were immunostained using antibodies against either EAAT4 or aldolase C (zebrin II), a protein known to exhibit the same para-sagittal banding pattern as EAAT4 (28). In both instances, the immunoreactivity was identical to that of the eGFP banding pattern (Fig. 2A), confirming that the eGFP fluorescence signal does accurately reflect EAAT4 expression profile and can be used to visually distinguish Z+ and Z– PC subtypes. Of note no difference in EAAT4, GLAST or GLT1 levels were observed between WT and WT-eGFP mice and neither full-length nor any truncated form of EAAT4 protein was detected in the cerebellum of ET4^–/–^-eGFP mice (Fig. 2B).

Having confirmed the fluorescent intensity of GFP accurately distinguishes PC subtypes, whole-cell patch clamp recordings were performed on either high GFP (Z+) or low GFP (Z–) expressing PCs in 6-week-old WT-eGFP, ET4^–/–^-eGFP and GLAST^–/–^-eGFP mice. This revealed that all ET4^–/–^ PCs exhibiting irregular burst firing (CV ISI > 1) were high GFP expressing (Z+) cells (Fig. 2C and D), whereas the regular spontaneous activity of low GFP expressing (Z–) ET4^–/–^ cells was not significantly different to that of WT cells (Z– WT, 41.6 ± 3.9 Hz; Z– ET4^–/–^, 48.6 ± 2.7 Hz; *P* = 0.66; Fig. 2C–E). In contrast, the majority of silent PCs in GLAST^–/–^ animals were low GFP (Z–) expressing cells and thus were PCs with low levels of EAAT4 protein (Fig. 2C). High GFP (Z+) expressing cells in GLAST^–/–^ mice were all observed to have a similar spontaneous intrinsic simple spike activity to that of WT cells (Z + WT, 39.8 ± 4.3 Hz; Z + GLAST^–/–^, 41.1 ± 8.3 Hz; *P* = 0.994; Fig. 2E).

Finally, although not all high GFP expressing (Z+) ET4^–/–^ cells were found to be bursting (CV ISI > 1) they exhibited a significantly higher tonic firing frequency (64.5 ± 9.4 Hz; *P* = 0.047) to that of Z+ WT cells (39.8 ± 4.3 Hz). Conversely, all the low GFP expressing (Z–) GLAST^–/–^ cells that were not silent were significantly less active, exhibiting a much lower firing frequency (9.6 ± 3.8 Hz; *P* = 0.001) to that of Z– WT cells (41.6 ± 3.9 Hz; Fig. 2E). Of note in 6-week-old WT animals a slightly higher firing frequency was detected in Z– WT cells compared with Z+ WT cells but a significant increase in firing rate was only observed in mice older than 3 months (Z+ 34.5 ± 2.9 Hz, *n* = 12; Z– 53.4 ± 7.5 Hz, *n* = 9; *P* = 0.04), an age similar to that used by Zhou and colleagues (37).

### mGluR1 activation downstream of EAAT4 loss mediates irregular firing of Z+ PCs

**Figure 3.**
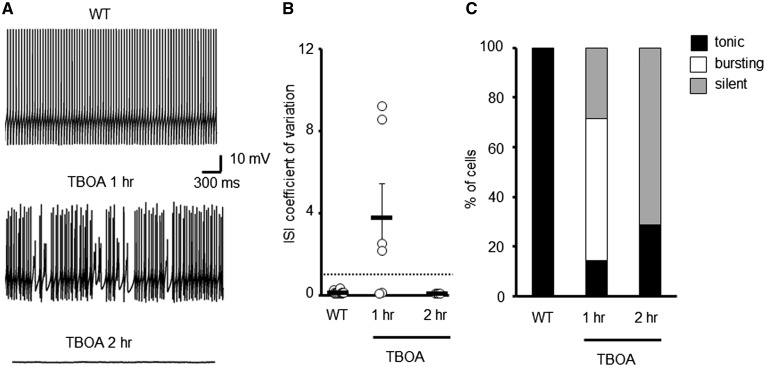
Acute elevations to extracellular glutamate levels alters spontaneous WT PC firing patterns. (**A**) Sample trace of WT PC and cells incubated with TBOA. (**B**) Coefficient of variation of ISI for WT PCs following pre-incubation with either vehicle (*N* = 3, *n* = 16) or TBOA (*N* = 3, *n* = 10). Dotted line depicts CV = 1. (**C**) Ratio of WT PCs that are tonically firing, bursting or are silent following pharmacological treatment to elevate extracellular glutamate levels.

To test whether an acute loss of transporter activity can alter spontaneous PC activity similar to a constitutive loss, WT cerebellar slices were pre-incubated with dl-TBOA (100 μM), a nonselective glutamate transporter antagonist prior to performing *in vitro* whole cell patch recordings. A concentration of 100 μM TBOA was chosen as it blocks a significant proportion of, but not all, transporter molecules and results in a physiologically relevant increase in ambient glutamate (39). Following pre-incubation for 1 h, the majority of PCs were found to exhibit an irregular pattern of firing (CV ISI 3.75 ± 1.67; Fig. 3), similar to that of high GFP expressing (Z+) ET4^–/–^ cells (2.9 ± 0.8; Fig. 2C and D). If incubation with dl-TBOA was extended to 2 h, further elevating ambient glutamate levels, then the majority of PCs were found to fall silent (Fig. 3A and C), with those not silent possessing a regular but lower frequency of firing (18.8 ± 1.04 Hz; CV ISI 0.06 ± 0.005; Fig. 3B). The pre-incubation with dl-TBOA was not pathological as normal tonic firing could be restored in all silent cells with a depolarizing current step.

**Figure 4.**
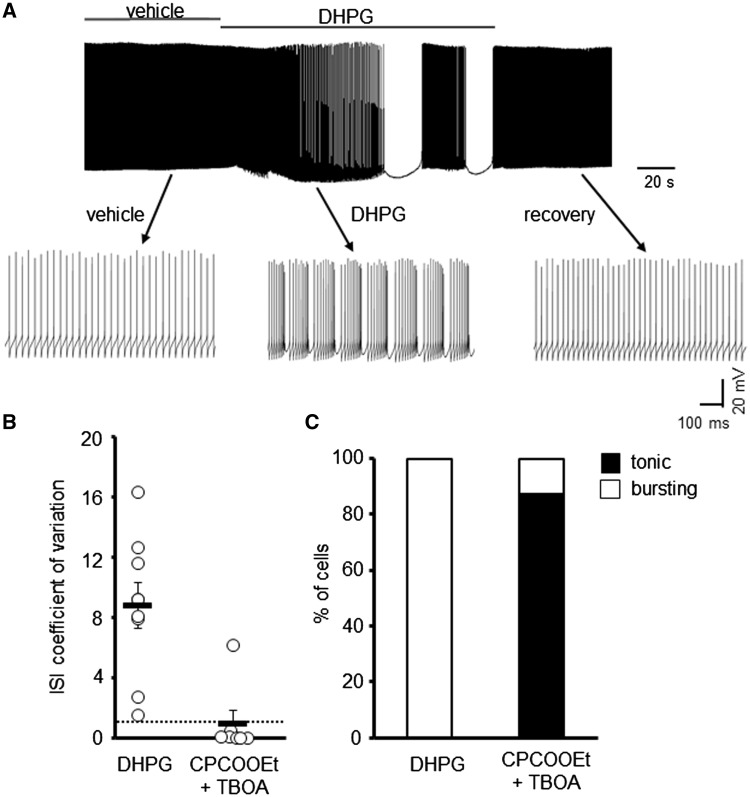
mGluR1 activation gives rise to irregular spontaneous WT PC firing. (**A**) Sample trace of WT PC in the presence and absence of DHPG (50 μM). (**B**) Coefficient of variation of ISI for WT PCs following application of DHPG (*N* = 4, *n* = 9) or pre-incubation with 10 μM CPCCOEt and 100 μM TBOA (*n* = 7). Dotted line depicts CV = 1. (**C**) Ratio of WT PCs that are tonically firing or bursting following pharmacological treatment to manipulate mGluR1 signalling.

Next we investigated the effect a group 1 mGluR agonist, dihydroxyphenylglycine (DHPG), had on the intrinsic firing properties of Z+ WT PCs. This pharmacological manipulation was carried out because regional differences in synaptic mGluR1α activity have been shown to correlate with the expression pattern of EAAT4 (31) and mGluR1 agonists were shown to induce burst activity in cultured Purkinje neurons (40). Excessive mGluR activation induced by the application of DHPG resulted in the irregular spontaneous firing of Z+ WT PCs (CV ISI 8.8 ± 1.5) but which was reversible following drug wash-out (Fig. 4A and B). Moreover, the TBOA-induced bursting phenotype in WT PCs was blocked in all but one cell by the co-incubation of TBOA for 1 h with the mGluR1 antagonist CPCCOEt (10 μM). The restoration of normal tonic firing by CPCCOEt (26.8 ± 5.5 Hz; CV ISI 0.14 ± 0.07) indicates that aberrant mGluR1 signalling was responsible for the irregular PC firing following elevated ambient glutamate levels (Fig. 4B and C).

### NMDA receptor activation downstream of GLAST loss abolishes Z– PC spontaneous activity

**Figure 5.**
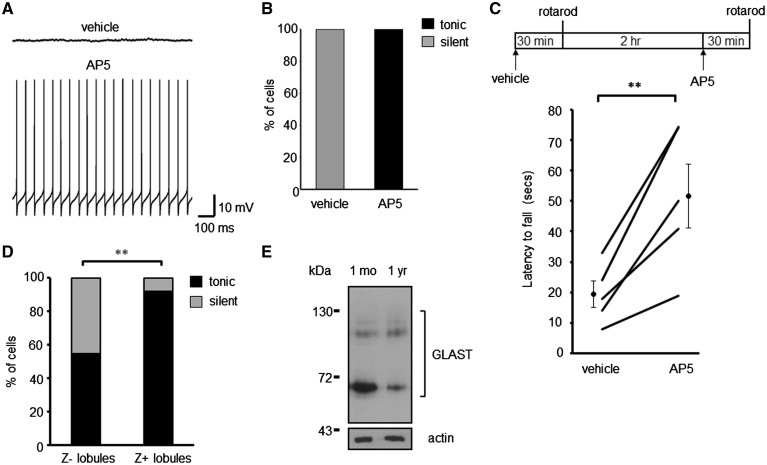
NMDA receptor blockade restores spontaneous PC firing in GLAST^–/–^ PCs and alleviates motor deficits. (**A**) Sample traces of Z– GLAST^–/–^ PCs pre-incubated with vehicle and with AP5 (50 μM). (**B**) Ratio of GLAST^–/–^ PCs that are silent or tonically firing following pharmacological treatment to block NMDA receptor activation. (**C**) Schematic of drug treatment paradigm and latency of GLAST animals to fall from rotarod at 10-rpm. Data following vehicle administration and 2.5 mg/kg AP5 is plotted for each animal, alongside the cohort mean ± SEM. (**D**) Ratio of modes of intrinsic activity in PCs from Z– and Z+ lobules in 1-year-old WT animals. (**E**) Immunoblot analyses of GLAST protein levels in cerebellar homogenates from 1-month and 1-year-old WT animals.

Depolarization-induced potentiation of inhibition has been reported in the cerebellum whereby activation of pre-synaptic NMDA (ionotropic glutamate) receptors on molecular layer interneuron terminals results in a sustained increase in inhibitory efficacy onto PCs (41). Furthermore, glial glutamate transporters have been shown to influence synaptic inhibition of PCs by limiting glutamate spillover and hence the activation of presynaptic NMDA receptors (42). At 6 weeks of age PCs are not believed to express functional NMDA receptors (43). We therefore determined whether excessive presynaptic NMDA activation could be responsible for the silencing of Z– PCs in GLAST^–/–^ mice due to glutamate spillover in regions of the cerebellar cortex with low EAAT4 levels. This was achieved by pre-incubating acute cerebellar slices from GLAST^–/–^-eGFP mice with AP5 (50 μM), an NMDA receptor antagonist, for 30 min prior to performing whole cell patch recordings. Following NMDA receptor blockade all Z– PCs in GLAST^–/–^ mice were no longer silent, but were found to be spontaneously active (21.2 ± 3.9 Hz; CV ISI 0.15 ± 0.04; *N* = 3, *n* = 8; Fig. 5A and B), in contrast to control slices where all silent PCs remained silent (*N* = 3, *n* = 13). We then investigated whether NMDA receptor blockade could rescue motor deficits in GLAST deficient mice by administering AP5 (2.5 mg/kg). The ability of animals to stay on the rotarod at 10 rpm following drug administration was compared with their pre-treatment performance (Fig. 5C). This revealed a significant improvement in motor performance following administration of AP5 (51.7 ± 10.5 s) compared with vehicle (19.4 ± 4.3 ; *P* = 0.0096 paired *t*-test; *N* = 5). There was no adverse effect of AP5 treatment on the motor behaviour of wild-type animals (vehicle, 120 s; AP5, 118.5 ± 1.5 s; *P* = 0.391 paired *t*-test; *N* = 4).

A similar effect on Z– PC spontaneous activity was observed with age. In 1-year-old WT mice 45.5% of PCs from the predominantly Z– anterior lobules (I–V) were found to be silent whereas only 8.3% of cells were silent in the predominantly Z+ posterior lobules (VIII–X) (*N* = 3, *n* = 22, lobules I–V; *N* = 5, *n* = 36, lobules VIII–X; *P* = 0.0023; Fig. 5C). This is in contrast to all Z+ and Z– PCs in young mice exhibiting regular spontaneous activity (Fig. 2C). A significant reduction in GLAST protein levels was observed in 1-year-old compared with 1-month-old WT mice (65.3 ± 1.9% of 1-month-old levels, *N* = 4, *P* = 0.0004; Fig. 5D) indicating the loss of GLAST protein could be a factor of age-associated silencing of Z– PCs and in turn age-related motor decline. These findings are also consistent with our previous study in a disease model of SCA type 5 where one third of *in vivo* PC recordings from lobule V in 8-month-old β-III^–/–^ mice exhibited no simple spike firing (5). At this age β-III^–/–^ mice have a significant reduction in GLAST protein (5) supporting the case that loss of GLAST function results in a decrease in PC activity and reduced output from the anterior cerebellar cortex.

### Differential effect of cerebellar transporter loss on PC survival

**Figure 6.**
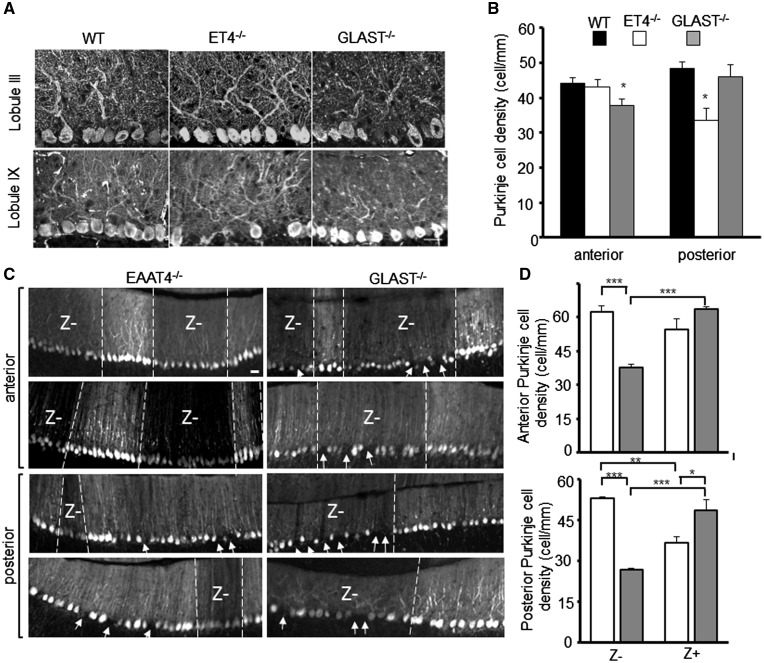
PC loss correlates with differential expression profile of EAAT4. (**A**) Representative confocal images of regions in lobule III and lobule IX from 1-year-old WT, ET4^–/–^ and GLAST^–/–^ mice. Midline sagittal cerebellar sections were immunostained with anti-ITPR1 antibody. Bar, 50 µm. (**B**) Quantification of anterior and posterior PC density in 1-year-old WT (*N* = 5), ET4^–/–^ (*N* = 3) and GLAST^–/–^ (*N* = 4) mice. (**C**) Representative images of regions from anterior and posterior coronal sections from 1-year-old ET4^–/–^-GFP and GLAST^–/–^-GFP mice with arrows indicating PC loss. Bar, 50 µm. (**D**) Quantification of posterior and anterior Z– and Z+ PC density in 1-year-old ET4^–/–^-GFP and GLAST^–/–^-GFP mice (*N* = 4). All data are means ± SEM.

Next we asked whether there was any regional difference in PC survival in ET4^–/–^ and GLAST^–/–^ animals. In mid-sagittal sections we found no difference in thickness of molecular layer between 1-year-old WT, ET4^–/–^ and GLAST^–/–^ animals (WT, 124 ± 2.9; ET4^–/–^, 122.8 ± 4.4; GLAST^–/–^, 121.2 ± 3.8 μm, *N* = 5) but a loss of PCs was observed in the anterior lobules of 1-year-old GLAST^–/–^ animals (*P* = 0.045) and in the posterior lobules of 1-year-old ET4^–/–^ mice (*P* = 0.04; Fig. 6A and B). In contrast, no PC loss was observed in either anterior or posterior lobules of 6-week-old ET4^–/–^ and GLAST^–/–^ animals (anterior ET4^–/–^ 106.7 ± 1.6, GLAST^–/–^ 97.6 ± 2.1, posterior ET4^–/–^ 104.3 ± 1.3, GLAST^–/–^ 102.6 ± 3.4% of WT). Upon examination of coronal sections from 1-year-old ET4^–/–^-GFP and GLAST^–/–^-GFP mice significant differences between high GFP expressing (Z+) and low GFP expressing (Z–) PCs was evident, with high and low GFP expressing PCs differentially lost in ET4^–/–^-GFP (posterior *P* = 0.005) and GLAST^–/–^-GFP mice (anterior *P* = 0.000079; posterior *P* = 0.001), respectively (Fig. 6C and D). In the case of GLAST^–/–^-GFP mice, the most profound loss of low GFP expressing PCs was out with the central vermis.

### EAAT4 knockout mice display progressive ataxia

Because this study revealed Z+ PCs in ET4^–/–^ mice exhibit irregular spontaneous intrinsic activity and with age there is loss of Z+ PCs, combined with previous studies that reveal EAAT4 loss affects synaptic events (7,44,45), we carried out a longitudinal behaviour analysis of ET4^–/–^ mice to determine whether they exhibit any motor deficits. This involved carrying out gait analysis, rotarod and an elevated beam task on 6-week, 6-month and 10-month-old WT and ET4^–/–^ animals.

**Figure 7.**
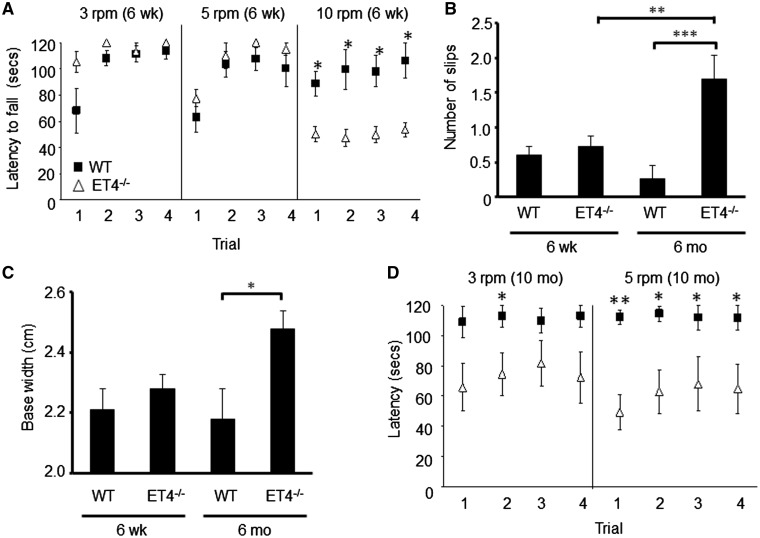
EAAT4 knockout mice exhibit progressive motor deficits. (**A**) Latency of 6-week-old animals to fall from rotarod at 3-, 5- and 10-rpm. (**B**) Number of hind-limb slips made by 6-week and 6-month-old mice when crossing narrow, elevated beam. (**C**) Hind-limb base width of 6-week and 6-month-old animals. (**D**) Latency of 10-month-old WT and ET4^–/–^ animals to fall from rotarod at 3- and 5-rpm. *N* = 14 (6-week WT), 22 (6-week ET4^–/–^), 12 (6-month WT), 10 (6-month ET4^–/–^), 5 (10-month WT) and 6 (10 month ET4^–/–^). All data are means ± SEM.

Young ET4^–/–^ animals (6 weeks of age) were significantly worse than WTs, on all four days of testing (*P* = 0.032, 0.016, 0.02, 0.01, days 1–4, respectively) when the speed of rotarod was 10-rpm, but not when tested at 3- or 5-rpm (Fig. 7A). Motor deficits in ET4^–/–^ animals were also found to be progressive as at 6 weeks of age, there was no difference in number of slips off the elevated beam (*P* = 0.996; Fig. 7B) or hind-limb base width (*P* = 0.997; Fig. 7C) but by 6 months of age ET4^–/–^ animals made more slips on the elevated beam (WT, 0.27 ± 0.19; ET4^–/–^, 1.7 ± 0.33, *P* = 0.0001; Fig. 7B), had a wider hindlimb base width (WT, 2.18 ± 0.1; ET4^–/–^, 2.48 ± 0.06 cm, *P* = 0.025; Fig. 7C) and by 10 months of age they were significantly worse on the rotarod at 5 rpm compared with age-matched WT animals on all days of testing (*P* = 0.006, 0.032, 0.035, 0.035, day 1–4, respectively; Fig. 7D).

### mGluR1 blockade restores regular PC firing and alleviates motor deficits in ET4^–/–^ mice

**Figure 8.**
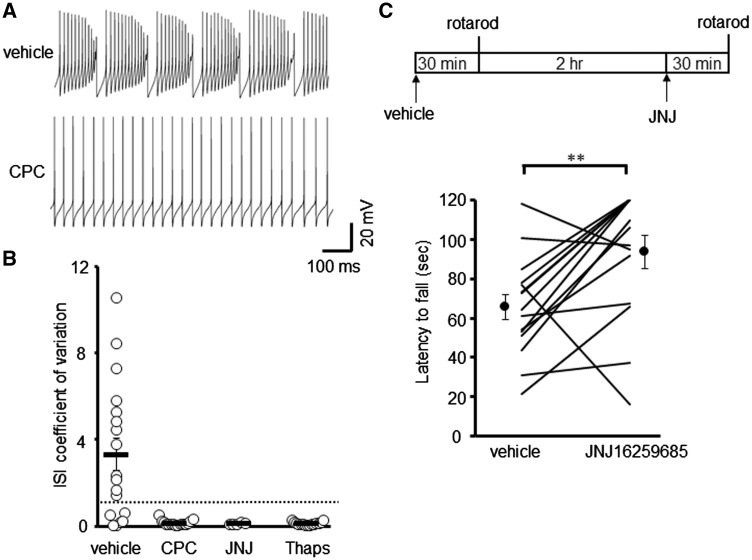
mGluR1 blockade restores regular PC firing and alleviates motor deficits in ET4^–/–^ mice. (**A**) Sample traces of Z+ ET4^–/–^ PCs pre-incubated with vehicle and with 10 μM CPCCOEt. (**B**) Coefficient of variation of ISI for ET4^–/–^ PCs following pre-incubation with either vehicle (*n* = 18), 10 μM CPCCOEt (CPC, *n* = 16), 3 nM JNJ16259685 (JNJ, *n* = 5) or 2 μM thapsigargin (Thaps, *n* = 15). Dotted line depicts CV = 1. (**C**) Schematic of drug treatment paradigm and latency of 6-week-old ET4^–/–^ animals to fall from rotarod at 10-rpm. Data following vehicle administration and 0.15 mg/kg JNJ16259685 is plotted for each animal, alongside the cohort mean ± SEM.

To test if mGluR1 blockade could restore regular firing in ET4^–/–^ mice, as was seen for the TBOA-induced bursting phenotype in WT PCs (Fig. 4A and B), acute cerebellar slices were pre-incubated with mGluR1 antagonists for 30 min prior to performing whole cell patch recordings. The irregular firing observed in high GFP expressing (Z+) ET4^–/–^ cells was abolished and tonic firing restored by both CPCCOEt (28.5 ± 2.6 Hz; CV ISI 0.14 ± 0.03) and JNJ16259685 (32.0 ± 3.8 Hz; CV ISI 0.10 ± 0.01), a selective and systemically active mGluR1 antagonist (Fig. 8A and B). Similarly pre-treatment with thapsigargin (2 µM), a Ca^2+^-ATPase inhibitor, to deplete IP_3_-gated Ca^2+^ stores restored tonic firing in ET4^–/–^ Z+ cells (26 ± 4.2 Hz; CV ISI 0.11 ± 0.02).

Next we investigated whether mGluR1 blockade could rescue the motor deficits observed in 6-week-old ET4^–/–^ mice by administering JNJ16259685 (0.15 mg/kg) (Fig. 8C and D). The ability of animals to stay on the rotarod at 10 rpm, the only motor task found to be impaired in young ET4^–/–^ mice, following drug administration was compared with their pre-treatment performance (Fig. 8D). This revealed a significant improvement in motor performance following administration of JNJ16259685 (93.7 ± 8.5 s) compared with vehicle (65.7 ± 6.5 s; *P* = 0.007 paired *t*-test; *N* = 15). No adverse effect of JNJ16259685 treatment was observed on rotarod performance of wild-type animals (118.3 ± 1.1 s; *P* = 0.724) but mice lacking GLAST were further impaired on the rotarod following administration of JNJ16259685 (5.9 ± 2.3 s; *P* = 0.011). Together the data indicate mGluR1 blockade can alleviate motor deficits resulting from loss of EAAT4, but not that of GLAST.

## Discussion

The pathogenic consequences of cerebellar glutamate transporter dysfunction have, to date, been largely related to excitotoxicity. Here we demonstrate for the first time that the two predominant cerebellar glutamate transporters, EAAT4 and GLAST/EAAT1, have critical but differential roles in the modulation of spontaneous PC firing patterns. This study expands our understanding of how glutamate transporter dysfunction through elevated extracellular glutamate and the dysregulation of peri- and extra-synaptic receptors, as well as receptors on nearby cells, contributes to disease pathogenesis in cerebellar disorders. Of note the study also highlights that in disease states the effect on cerebellar cortical output from specific PC populations and hence inhibition onto neurons in the cerebellar nuclei will differ depending on the EAAT dysfunction. High levels of EAAT4 protein are required to constrain the inherent excitability and plasticity of Z+ PCs by limiting mGluR1 signalling. In contrast GLAST/EAAT1 appears to play no role in modulating the firing pattern of the Z+ subpopulation of PCs but it does have a fundamental role in sustaining normal spontaneous simple spike activity in low EAAT4 expressing (Z–) PCs by preventing NMDA receptor activation. Loss of GLAST from these regions of the cerebellum results in a substantial decrease in spontaneous activity of Z– PCs, with the majority falling silent and the other Z– cells exhibiting a very low intrinsic firing frequency. In addition, both these cerebellar transporters were found to have differential effects on PC survival, with EAAT4 expression important in preventing Z+ PC death in the posterior cerebellum, whereas GLAST expression is required for the long-term survival of PCs expressing low levels of EAAT4. Together the data provide strong evidence that the consequence of GLAST/EAAT1 or EAAT4 loss varies between cerebellar regions, but in both cases it has a profound effect on intrinsic properties of PCs, cell survival and motor coordination. This new knowledge that ambient elevations in extracellular glutamate due to glutamate transporter dysfunction can alter the spontaneous firing pattern of PCs contributes to a better understanding of disease mechanisms.

### Loss of EAAT4 results in ataxia and selective PC death

Here we show for the first time that loss of EAAT4 results in an ataxic phenotype and that the motor deficits are progressive. Although it has previously been reported that EAAT4 knockout mice do not have a cerebellar phenotype, this observation was simply based on a visual inspection of cage behaviour (46). Without direct assessment of motor task function motor deficits are likely to be overlooked; indeed given the young age of ET4^–/–^ mice studied it is also possible that the visual observation of cage behaviour was not carried out on sufficiently aged ET4^–/–^ animals (46).

This study is also the first to implicate loss of EAAT4 in PC death and specifically of Z+ cells in the posterior cerebellum. Previous studies using ET4^–/–^ mice either did not look (44,45) or analysis was not carried out on aged animals (47). Furthermore in the latter study using 8- to 12-week-old mice PC density was only quantified within the anterior cerebellum (lobule III) and not posterior lobules. The authors report PC loss following brain ischemia in lobule III of GLAST knockout mice but not EAAT4 knockout mice (47). This is in agreement with our findings but we also find that low EAAT4 expressing PCs in the posterior cerebellum, especially out with the central vermis, are vulnerable to cell death in the absence of GLAST protein.

Therefore, in studies of ataxia where a loss of EAAT4 is observed this can now be directly implicated in a motor phenotype, so contributing to a better understanding of mechanisms that underpin cerebellar ataxia. The defects in cerebellar cortical output would likely arise from a combination of effects following EAAT4 loss; notably the findings reported here that Z+ PCs in the posterior cerebellum exhibit irregular firing patterns due to aberrant mGluR1 signalling and altered intrinsic properties and that they are more vulnerable to cell death following EAAT4 loss but also that EAAT4 loss affects synaptic events, altering the size and/or duration of excitatory postsynaptic currents at parallel fibre-PC synapses (7,44,45) and impairing the clearance of released glutamate at climbing fibre-PC synapses (46,48,49). Of note the latter defect is confounded by the fact that greater glutamate release was shown to occur from climbing fibre terminals in zebrin-positive regions (50).

### Intrinsic Z+ PC membrane firing pattern is regulated by EAAT4 and mediated by mGluR1 signalling

Our data suggest that the irregular firing of Z+ PCs and motor incoordination resulting from EAAT4 loss are mediated by mGluR1 signalling. This is consistent with other recent studies that have implicated dysregulated mGluR1 signalling in altered PC excitability and ataxia. For example, prolonged mGluR1 activity at cerebellar parallel fibre–PC synapses was observed in moderately ataxic SCA1 mice (51) and mGluR1 activation was also shown to increase intrinsic PC firing in cerebellar slices from SCA2 mice (52). Moreover, consistent with our findings in regard to mGluR1 antagonist improvement in motor performance in ET4^–/–^ mice, studies show that modulating mGluR1 activity and glutamatergic stimulation can restore motor performance in other mouse models of ataxia (51,53). Removing excessive mGluR1 activity by treating with JNJ16259685 improved motor performance in young SCA1 (51) but not old SCA1 mice (54). This difference in response to mGluR1 modulation may be due to the subsequent loss of GLAST in later stages of disease in SCA1 mice (4) as we find that mGluR1 blockade further impairs motor performance in mice lacking GLAST. In SCA28 mice reducing mGluR1 expression or increasing EAAT2 expression does alleviate ataxia (53). Of note, intrinsic excitability of wild-type PCs was found to be increased following induction of long-term depression in the parallel fibre-PC synapses (55), an event dependent on mGluR1 activity (56).

Currently it is unclear what downstream PC dendritic ionic conductance(s) are affected and responsible for the changes in neuronal firing observed in this study. However, deciphering the complex interplay of metabotropically regulated currents will be essential in elucidating the downstream cellular mechanisms that underpin alterations to PC intrinsic properties and which contribute to aberrant cerebellar output in disease states. Possibilities include the activation of low-threshold T-type calcium channels (57), changes to the surface expression of HCN channels, regulated by PLCβ/IP_3_R and protein kinase C activities (58), or differences in potassium channels some of which have been implicated in ataxia (59–62). Whether the differential expression of isoforms of mGluR1 (63), PLC beta (64) or other downstream signalling molecules such as αCaMKII are important in the regional effects mediated by the expression profile of cerebellar EAATs remains to be elucidated. Given mGluR1b does not interact with homer proteins (65) and is complementary to zebrin II (63), hence EAAT4, it could be that differences in the postsynaptic effectors of homer-dependent and homer-independent mGluR1 isoforms are relevant in the differential regulation of PC intrinsic properties within parasagittal compartments. For instance mGluR1a possesses higher affinity and coupling efficacy to G proteins and phospholipase C than mGluR1b (66). This would result in differences in Ca^2+^ release from intracellular stores and may account for Z+ PCs reacting differently to that of Z– PCs following loss of EAAT function. The differences in glutamate release (50) and calcium signalling downstream of mGluR1a activation may also account for the fact that the proximal dendrites, sites of climbing fibre innervation, are the first to degenerate in PCs from the posterior cerebellum of β-III^–/–^ mice that lack functional EAAT4 (5,7).

In the future identifying all the proteins that underlie the different physiological profiles of PC subpopulations will be key to understanding the functional diversity of microzones within the cerebellar cortex and how distinct changes to firing patterns occur in different disease states.

### Modulation of Z– PC intrinsic properties by GLAST/EAAT1 activity as a potential factor in neurological disorders

Physiological defects have been reported to occur from changes to GLAST/EAAT1 activity with GLAST knockout mice exhibiting progressive motor incoordination (7,67) and mutations in *SLC1A3*, the gene encoding EAAT1, being associated with episodic ataxia type 6 (EA6) (9–11). In the case of one EA6 point mutation, substitution of proline 290 by an arginine, there is both an abnormal chloride conductance and a decrease in glutamate transport rates (9,68). A recent study in *Drosophila* proposes it is excessive chloride extrusion by the mutant transporter that is relevant to disease pathogenesis, as increased chloride uptake rescues locomotor performance (69). It is proposed that changes to intracellular chloride concentrations affect the osmotic balance, reduce cell volume and therefore ensheathment of synapses by astrocytes (69). A recent study also reports a retraction of Bergmann glial processes from PC dendrites in GLAST knockout mice (70). Such a reduced glial coverage of synapses superimposed on loss of GLAST/EAAT1 would further facilitate glutamate diffusion, extra-synaptic crosstalk and altered ionic conductances within dendrites. Together these studies improve our understanding of how PC dysfunction can arise following loss of the glial glutamate transporter GLAST in regions where EAAT4 levels are low. Given NMDA receptors composed of GluN1/GluN2C and GluN1/GluN2D subunits are less sensitive to Mg^2+^ block (71) it is likely that these are the molecular layer interneuron receptor subtypes being activated by glutamate spillover. Furthermore as all recordings were performed in the presence of picrotoxin, a GABA_A_ receptor antagonist it is proposed that aberrant dendritic GABA_B_ receptor activation is involved, providing insights into the cellular mechanisms downstream of loss of GLAST function that underpin cerebellar dysfunction. This is consistent with the finding that baclofen, a GABA_B_ agonist inhibits PC firing and that this inhibition persists in the presence of a GABA_A_ antagonist (72).

Conversely increased EAAT1 activity has been suggested to be a factor in some neurodevelopmental disorders. A functional *SLC1A3* variant has been reported to be more prevalent in individuals with Tourette syndrome than controls and found to result in increased EAAT1 membrane insertion and glutamate uptake (73). A gene duplication of *SLC1A3* has also been reported as a possible risk factor for autism and attention deficit/hyperactivity disorder–like behaviour (74). The current study therefore points towards altered EAAT1 glutamate transport activity being implicated in neurodevelopmental disorders by modulating dendritic ionic conductances and hence spontaneous PC activity. In fact similar regional cerebellar differences to that observed in GLAST^–/–^ and ET4^–/–^ mice have been reported in Shank2-deficient mice that exhibit autism-like behaviour (75). A significant decrease in simple spike frequency was observed in anterior lobules of global Shank2^–/–^ mice, but not in their posterior lobules whereas a consistent irregularity of simple spikes was observed in PCs from the posterior but not anterior lobe. Depolarizing current steps have also revealed that PCs in the posterior, but not the anterior, cerebellum of β-III^–/–^ mice exhibit increased membrane excitability compared with wild-type PCs (data not shown) even though they display reduced spontaneous simple spike activity due to loss of ankyrin R and lower densities of Na_v_1.1 and Na_v_1.6 (5,76). Similarly GLT1/EAAT2 dysfunction has been implicated in enhanced pyramidal neuronal excitability in fragile X syndrome (77) and pathological repetitive behaviours (78). It therefore appears that alterations to distinct physiological profiles and intrinsic activity of PCs within microzones of the cerebellar cortex downstream of aberrant EAAT activity impairs the correct processing and efficient transmission of information possibly by decoupling coordinated activation of neighbouring functional microzones (79). Future studies are needed to address how aberrant cerebellar EAAT activity modulates intrinsic PC dendritic properties, not only in disorders of the motor system, but also in autism spectrum disorders and other cognitive and neuropsychiatric disorders given the emerging role of cerebellar dysfunction in these disorders (18–23).

To conclude, this study identifies a new and central role for plasma membrane glutamate transporters in regulating spontaneous PC activity and survival, both key determinants of cerebellar output, by restricting the activation of peri- and extra-synaptic receptors, as well as receptors on nearby cells. Consequently altering functional cerebellar EAAT levels may prove very effective in restoring dendritic ionic conductances and neuronal function in a number of neurological disorders where intrinsic neuronal excitability is altered.

## Materials and Methods

### Animals

All procedures involved in generation and analysis of mutant mice were carried out according to the United Kingdom Animals (Scientific Procedures) Act (1986) and other Home Office regulations under specific-pathogen-free conditions. GLAST^–/–^ and ET4^–/–^ mice, both on a C57BL/6 genetic background, were maintained and crossed with eGFP-reporter mice to generate GLAST^–/–^-eGFP and ET4^–/–^-eGFP mice. The genotypes of all animals were determined by PCR analysis on genomic DNA extracted from ear notch biopsies using ChargeSwitch gDNA tissue kit (Invitrogen, Carlsbad, CA) and both sexes were used in all experiments. For ET4^–/–^ mice a common upstream primer (5′-ttcctgattgctggaaagattctgg-3′) and primers specific for the wild-type allele (5′-agttcagggaaaggccataccttgg-3′) and the *PGK-neo* cassette in the mutant allele (5′-ggatcggccattgaacaagatgg-3′) were used for amplification at an annealing temperature of 57°C. The 220-bp (from wild-type allele) and 1200-bp (from targeted allele) PCR products were resolved by electrophoresis on a 1.6% (w/v) agarose gel. For GLAST^–/–^ mice the specific primer sets used for amplification of wild-type allele were (5′-aagtgcctatccagtccaacga-3′; 5′-aagaactctctcagcgcttgcc-3′) and mutant allele (5′-aatggaaggattggagctacgg-3′; 5′-ttccagttgaaggctcctgtgg-3′) at an annealing temperature of 54°C. The 214-bp (from wild-type allele) and 362-bp (from targeted allele) PCR products were resolved by electrophoresis on a 1.6% (w/v) agarose gel. The primer set specific for GFP was 5′-aagttcatctgcaccaccg-3′and 5′-tccttgaagaagatggtgcg-3′and used at an annealing temperature of 55°C. The 220-bp GFP PCR product was resolved by electrophoresis on a 1.6% (w/v) agarose gel. Immunoblot analysis using polyclonal antibody against EAAT confirmed ET4^–/–^ genotype in ET4^–/–^-eGFP mice (presence of EAAT4-BAC construct prevented detection of homozygosity by PCR). All knockout mice were viable although pups from GLAST^–/–^ and GLAST^–/–^-eGFP mice were routinely fostered with CD1 mothers to ensure survival.

### Immunoblotting

Whole cerebella were homogenized in 400 μl of ice-cold homogenization buffer [20 mM HEPES, pH 7.4, 1 mM EDTA, 1 mM phenylmethylsulfonyl fluoride and protease inhibitor cocktail (Calbiochem, San Diego, CA)] with a Teflon-glass homogenizer. Protein concentrations were determined using Coomassie-Plus Reagent and bovine serum albumin as standard (Pierce, Rockford, IL). Protein samples were resolved by denaturing SDS-polyacrylamide gel electrophoresis and transferred to nitrocellulose membranes (Amersham Pharmacia). The membranes were blocked for 1 h at room temperature with 5% (w/v) non-fat dry milk in Tris-buffered saline/Tween 20 [TBS/T, 20 mM Tris, 200 mM NaCl (pH 7.6) with 0.1% (v/v) Tween-20]. Blots were incubated overnight at 4°C with either rabbit anti-EAAT4, -GLAST (1:200), -GLT1 (1:4000) (all from Jeffrey Rothstein), or mouse anti-actin (1:1600; Sigma, St. Louis, MO) in blocking buffer. After washing with TBS/T the blots were incubated for 1 h at room temperature with either HRP-conjugated donkey anti-rabbit IgG, or HRP-conjugated sheep anti-mouse IgG (1:4000; Amersham Pharmacia). Immunoreactive proteins were visualized with enhanced chemiluminescence (ECL, Santa Cruz Insight Biotechnology, Wembley, UK).

### Immunohistochemistry

Brains were removed and immersion-fixed with 4% (w/v) paraformaldehyde in 0.1 M sodium phosphate buffer, pH 7.4 overnight at 4°C and cryoprotected by immersion in 0.1 M sodium phosphate buffer (pH 7.4) containing 30% (w/v) sucrose. 30-μm-thick coronal or mid-sagittal free-floating cerebellar sections were incubated for at least 3 h with blocking solution [5% (v/v) normal goat serum with 0.4% (v/v) Triton X-100 in PBS] prior to applying rabbit anti-EAAT4, anti-aldolase C and anti-ITPR1 antibody (Millipore) [2% (v/v) normal goat serum/0.1% (v/v) Triton X-100 in PBS] overnight at 4°C. Sections were washed three times in PBS before applying either Cyanine 3 (Cy3)-conjugated goat anti-rabbit IgG (Jackson laboratories) or fluorescein isothiocyanate–conjugated goat anti-rabbit IgG (Cappel) for 1 h at room temperature followed by three rinses in PBS and coverslipping with Vectashield (Vector Laboratories, Burlingame, CA). All quantification was carried out blind to genotype and involved counting the number of PCs, along 1 mm linear lengths in anterior (II–IV) and posterior (VIII–X) lobules from three sections/animal and the counts averaged for each animal. Images were captured with either a Zeiss inverted LSM510 or Nikon confocal laser scanning microscope in the IMPACT imaging facility, Centre for Discovery Brain sciences. Colours were applied using Image J.

### Slice electrophysiology

Cerebella (from 6-week-old animals unless otherwise stated) were dissected out into ice-cold modified artificial cerebrospinal fluid (ACSF) containing (in mM): 60 NaCl, 118 sucrose, 26 NaHCO_3_, 2.5 KCl, 11 glucose, 1.3 MgCl_2_ and 1 NaH_2_PO_4_ at pH 7.4 when bubbled with 95% O_2_:5% CO_2_. The cerebellar vermis was glued to the vibratome cutting platform (Leica VT 1200S, Germany) with cyanoacrylate adhesive. About 200 µm-thick sagittal slices cut from the central vermis were incubated for 30 min at 30°C in standard ACSF composed of the following (in mM): 119 NaCl, 2.5 CaCl_2_, 26 NaHCO_3_, 2.5 KCl, 11 glucose, 1.3 MgCl_2_ and 1 NaH_2_PO_4_ at pH 7.4 when bubbled with 95% O_2_:5% CO_2_, followed by 1 or 2 h 100 μM TBOA (dl-*threo*-*β*-benzyloxyaspartic acid), 30 min 10 μM CPCCOEt, 3 nM JNJ16259685, 2 μM thapsigargin, 50 μM AP5 or wash-in of 50 μM DHPG. Slices were transferred to a submerged recording chamber and superfused with standard ACSF (3–5 ml min^−1^) at 32°C with 10 μM NBQX and 50 μM picrotoxin to block AMPA and GABA_A_ receptors, respectively. PCs were visualized with a 40× immersion objective and Normarski differential interference contrast optics. Whole-cell recordings were obtained from PCs using thick-walled borosilicate glass pipettes with resistances of 3–5 MΩ. The internal solution contained (in mM): 125 K-Gluconate, 15 KCl, 10 HEPES, 5 EGTA, 2 MgCl_2_, 0.4 NaGTP, 2 NaATP and 10 Na-phosphocreatine, adjusted to pH 7.4 with KOH. All PCs showing spontaneous activity were routinely recorded for 5–10 min. For those cells that were silent or were burst firing current was injected at least 2 min into the recording. Data were acquired using pClamp 9 (Molecular Devices, Sunnyvale, CA) and recordings were filtered at 2 kHz and sampled at 10 kHz. Data analysis was carried out in Neuromatic, IGOR Pro (Wavemetrics, Lake Oswego, OR) and using in-house MatLab scripts.

### Motor coordination tests

Footprint patterns were analyzed using a runway (80 cm by 10.5 cm wide) with white paper at the bottom. Hind paws of animals were dipped in non-toxic, water-soluble black ink (Indian Ink, Winsor & Newton, Harrow, UK). Three consecutive strides were measured for each animal and base width was measured as the distance between the centre of one paw print to the centre of the next print on the opposite side. The elevated beam test was performed using a narrow horizontal beam (2 cm wide, 80 cm long, held at a height of 30 cm from the table). The number of hind paw slips the animal made whilst traversing the beam were counted. In the rotarod test the time a mouse remained on a rotating (3, 5 and 10 rpm) 3-cm-diameter cylinder was recorded (maximum 120 s; four trials per speed; TSE Rotarod, Bad Homburg, Germany). 6-week-old ET4^–/–^ mice received intraperitoneal injections of vehicle (sterile saline with 10% (w/v) hydroxypropyl-betacyclodextrin) 30 min before rotarod testing. Two hours after completion of rotarod testing, the same mice were injected with 0.15 mg/kg JNJ16259685 (Tocris Bioscience) dissolved in vehicle. Administration of JNJ at higher doses resulted in greater passivity, reduced rearing, a crouching behaviour and impaired motivation. About 6-month-old GLAST deficient mice received intraperitoneal injections of vehicle (sterile water) 30 min before rotarod testing. Two hours after completion of rotarod testing, the same mice were injected with 2.5 mg/kg AP5 (Tocris Bioscience) dissolved in vehicle.

### Statistics

Statistical analysis was carried out using SPSS v21 (SPSS Inc., Chicago, IL, USA). Analysis was performed using Student’s *t*-test for two groups of data and one-way analysis of variance for comparisons of three or more data groups. Contingency table and Fisher’s tests were used for categorical data evaluation. Significance was accepted at *P*-values < 0.05. *N* indicates the number of animals and *n* indicates the number of cells.
